# Eosinophil diversity and function: emerging insights into subtypes and therapeutic targets

**DOI:** 10.1038/s41420-026-03234-4

**Published:** 2026-07-10

**Authors:** Faiz Ali Khan, Abhishek Raj, Hizbullah Khan, Mammat Nurahmat, Maimaitijiang Abuduwake, Naibijiang Maitusun, Jingcheng Dong

**Affiliations:** 1 Institutes of Integrative Medicine, Fudan University, Shanghai, China; 2 Department of Integrative Medicine, Huashan Hospital, Fudan University, Shanghai, China; 3 Guangdong Provincial Key Laboratory of Medical Immunology and Molecular Diagnostics, School of Medical Technology, Guangdong Medical University, Dongguan, China; 4 Xinjiang Key Laboratory of Hetian Characteristic Chinese Traditional Medicine Research, Xinjiang Hetian College, Hetian, China

**Keywords:** Mucosal immunology, Cell death and immune response, Asthma, Prognostic markers, Acute inflammation

## Abstract

Eosinophils are multifunctional polymorphonuclear granulocytes garnering attention for their roles in disease and maintaining physiological homeostasis. Historically, these immune cells were known to mediate allergic responses and defend against parasitic infections. Recent evidence further links eosinophils to several eosinophil-associated disorders, including eosinophilic esophagitis, eosinophilic granulomatosis with polyangiitis, and hypereosinophilic syndrome; however, their pathogenic contribution appears to be disease-specific and remains incompletely defined in some contexts. The advances in immunology and molecular biology have yielded targeted biologic therapies against eosinophils, significantly improving outcomes for patients with eosinophilic disorders. Concurrently, research reveals that eosinophils are a heterogeneous cell population comprising distinct subtypes that differ in surface protein expression, tissue localization, and functional properties. Specifically, resident eosinophils (rEos) reside in tissues for local maintenance and immune regulation, whereas inflammatory eosinophils (iEos) are rapidly recruited during immune responses to drive inflammation. Initially characterized in murine models, similar eosinophil heterogeneity has since been confirmed in human blood and tissues. Recognizing distinct eosinophil subsets is crucial for understanding their contributions to disease progression. This review highlights recent discoveries in eosinophil diversity and explores therapeutic implications of selectively targeting specific eosinophil subsets in clinical practice.

## Facts

- The specific molecular mechanisms that define the functional dichotomy between homeostatic rEos and iEos in different human tissues and diseases remain a major subject of research.
- The existence and clinical relevance of the rEos and iEos subsets defined in mice must be conclusively validated in human pathophysiology to translate preclinical findings.
- A key therapeutic challenge is the development of subset-specific biologics that selectively deplete pathogenic iEos while sparing the homeostatic functions of rEos.
- A major gap is confirming whether the rEos/iEos paradigm established in mice defines functionally distinct and clinically relevant populations in humans.
- Eosinophil heterogeneity may define disease endotypes, where the relative abundance of rEos and iEos could explain differential treatment responses.

## Open questions

- How can we therapeutically target pathogenic iEos while preserving the homeostatic functions of rEos to advance the treatment of eosinophil-associated diseases?

## Introduction

Granulocytes in peripheral blood were first formally delineated in 1846 by Jones [1] and Brewer [2], whose morphological and functional characterization laid the groundwork for subsequent hematological and immunological research. Because of the lack of effective staining techniques at the time, they could not accurately distinguish the granulocyte subtypes. This limitation was later overcome in 1879, when Paul Ehrlich introduced an aniline dye-based blood-staining method using coal-tar dyes such as eosin [3]. He successfully identified eosinophils and named them by their unusual affinity for the eosin dye. These spherical cells, measuring 12–17 micrometers in diameter, are characterized by a bilobed nucleus and cytoplasm that stains pink due to their acidophilic properties and strong affinity for eosin. Functionally, they are classified as nonspecific cytotoxic cells that participate in destructive processes. Their primary origin lies in pluripotent hematopoietic stem cells within the bone marrow, which differentiate through defined progenitor stages into the mature eosinophilic lineage. In some cases, however, these cells may also develop in the thymus or spleen [1]. Furthermore, he not only characterized the distinct staining properties of eosinophil granules but also examined their distribution across various species and tissues. From these studies, he hypothesized that eosinophils develop in the bone marrow—a prescient insight consistent with current understanding of eosinophil hematopoiesis.

**Fig. 1:**
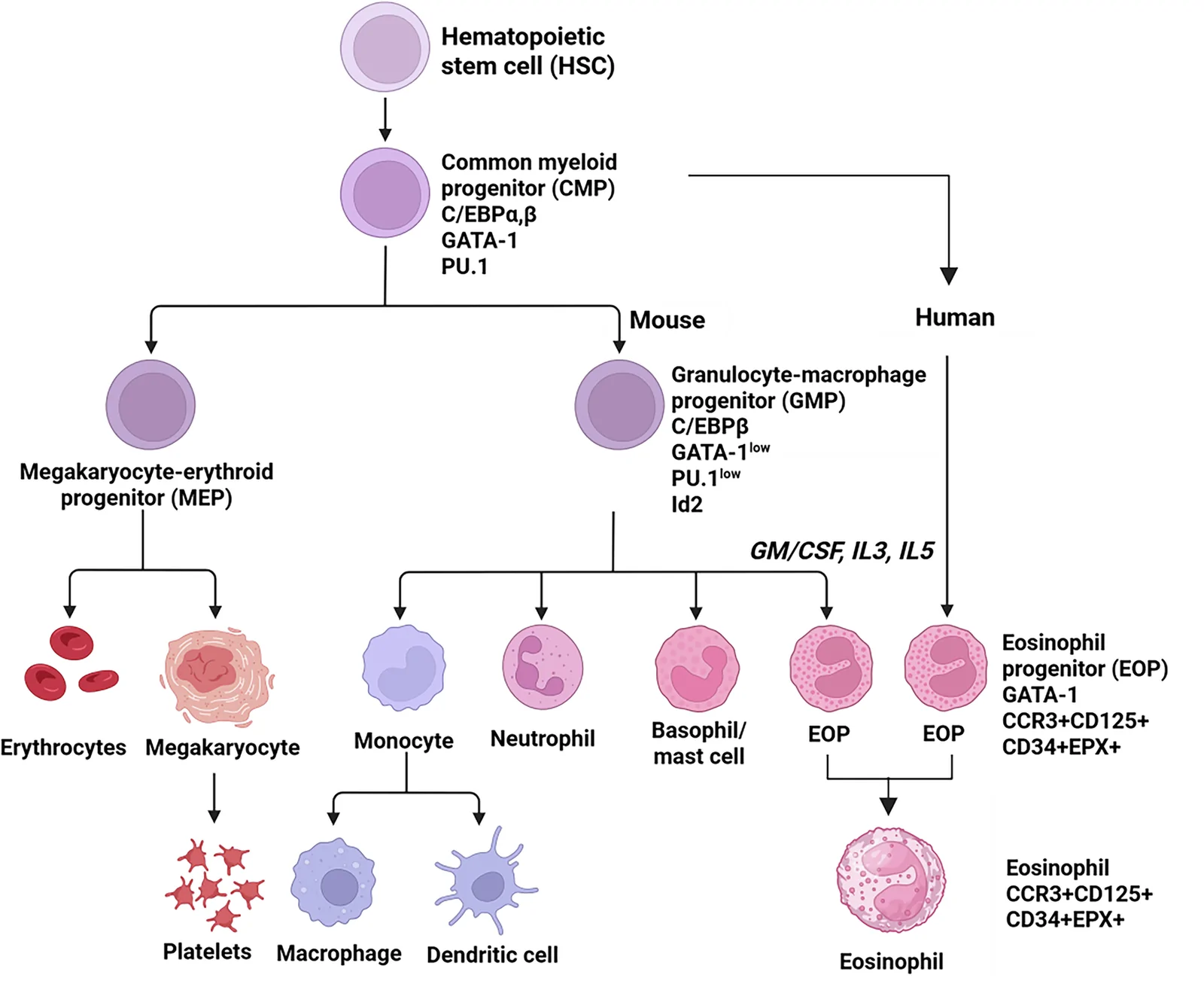
Hierarchical tree of eosinophil progenitor formation in the bone marrow. CMPs give rise to MEPs and GMPs, with eosinophil commitment regulated by C/EBP, GATA-1, and modulated by PU.1, FOG, and Id1. Eosinophil progenitors expand under GM-CSF, IL-3, and IL-5. Later on, these cells downregulate CD34 and leave the bone marrow for the circulation. Notably, human eosinophil progenitors arise directly from CMPs, whereas in mice they develop via GMPs, which are bipotent for eosinophils and neutrophils.

Identifying the precursor of the eosinophil took several more decades. In 1960, Rytömaa observed that eosinophils were especially abundant in the bone marrow, reinforcing the idea that their origin lies there. It was not until 1984 that Fischkoff et al. provided definitive evidence of a common progenitor: they demonstrated that eosinophils and neutrophils arise from a common precursor cell using the promyelocytic cell line HL-60 as a model [4]. This finding established that eosinophils share a developmental lineage with other granulocytes. Molecular advances in the late 20th century further clarified eosinophil lineage commitment. The genetic and epigenetic regulation of eosinophils, with particular emphasis on the critical roles of the transcription factor GATA-1 and interleukin (IL)-5, is under active investigation, highlighting important therapeutic implications [5]. In 1998, a gene selectively expressed in developing eosinophils, EOS47, was identified; its promoter region contains binding sites for key hematopoietic transcription factors such as Myb-Ets, C/EBP, and GATA [6], which are crucial for lineage specification. Consistent with this, subsequent studies showed that eliminating a high-affinity GATA-1 binding site in the GATA-1 gene promoter abolishes the eosinophil lineage [7], highlighting GATA-1’s essential role in eosinophil differentiation (Fig. 1).

Parallel research uncovered how the immune system regulates eosinopoiesis (the production of eosinophils). It has been observed that immunocompetent lymphocytes are responsible for the elevated eosinophil counts in peripheral blood during parasitic infection [8]. These findings suggested that factors released by lymphocytes stimulate eosinophil expansion as part of the immune response to parasites. A few years later, one such factor was identified: interleukin-5 (IL-5), the key cytokine driving eosinophil development. IL-5 was found to promote the terminal differentiation of eosinophils in the bone marrow and to enhance their proliferation and activation, and prolong their survival by inhibiting apoptosis [9].

Collectively, these landmark discoveries have shaped the current understanding of eosinophil biology. Each breakthrough—from the first identification of eosinophils to the elucidation of their molecular regulators – has provided fundamental insights into how eosinophils develop and function. These insights now form the basis for modern clinical perspectives, guiding research and therapy in diseases where eosinophils play a prominent role.

## Granule dynamics and exocytosis

Eosinophils are granulocytes with numerous cytoplasmic granules that store a variety of proteins, cytokines, chemokines, enzymes, and lipid mediators essential to their function [10]. A defining feature of eosinophils is the presence of four major cationic proteins within their secondary granules: major basic protein 1 and 2 (MBP1, MBP2), eosinophil peroxidase (EPO), eosinophil cationic protein (ECP), and eosinophil-derived neurotoxin (EDN). When released, these granule proteins can induce significant tissue damage, bronchoconstriction, mucus hypersecretion, and dysfunction [11]. The accumulation of eosinophils in tissues and their detection in biofluids are strongly correlated with the severity of eosinophilic disease.

Each of these eosinophil-specific granule proteins has potent biological activities. ECP and EDN are ribonucleases with antiviral properties; notably, ECP can form voltage-insensitive toxic ion pores in target cell membranes, potentially facilitating the entry of other cytotoxic molecules [12]. In addition to its cytotoxic actions, ECP also exhibits several immunomodulatory effects, including suppression of T-cell proliferation, inhibition of B-cell immunoglobulin production, induction of mast cell degranulation, and stimulation of airway mucus secretion and fibroblast glycosaminoglycan production [13]. MBP, another abundant granule protein, can alter smooth-muscle contractility by dysregulating vagal M2 and M3 muscarinic receptor function, and it triggers the degranulation of mast cells and basophils [14]. EPO constitutes roughly 25% of the total protein content of eosinophil granules. This enzyme catalyzes the oxidation of pseudohalides and nitric oxide to generate highly reactive oxygen species (e.g., hypohalous acids) and reactive nitrogen species like peroxynitrite, which in turn oxidize nucleophilic targets in proteins [15, 16]. These oxidative reactions promote cellular oxidative stress and can lead to target cell death via apoptosis or necrosis [15, 16].

Upon appropriate stimulation, eosinophils can release their granule contents by four primary mechanisms: classical exocytosis, compound exocytosis, piecemeal degranulation, and cytolysis. In classical exocytosis, individual granules fuse directly with the plasma membrane, releasing their entire contents into the extracellular space. Compound exocytosis is a variant wherein multiple granules first fuse together inside the cell, and then this larger composite granule fuses with the plasma membrane to secrete its contents [17]. Piecemeal degranulation (PMD) is a form of regulated secretion in which granule-derived mediators are selectively mobilized in small packets via transport vesicles rather than full-granule release, allowing for a gradual and controlled secretion of specific substances [17].

The fourth mechanism, eosinophil cytolysis (ECL), involves lytic cell death and rupture of the eosinophil via planned non-apoptotic cell death [18, 19]. There is evidence that eosinophil degranulation and ECL play a role in human disease from studying the ultrastructure of tissue biopsies, staining for extracellular granule proteins, and finding soluble granule proteins in different clinical samples [19, 20]. Although PMD and ECL make up most of the protein released from granules in patients, ECL also triggers inflammatory signals via chromatolysis, extracellular dsDNA traps, and pro-inflammatory galactin-10-containing Charcot–Leyden crystals [21, 22]. During cytolysis, the cell undergoes chromatolysis (disintegration of nuclear chromatin) followed by plasma membrane rupture, leading to the release of intact, membrane-bound granules into the extracellular space – these released granules are known as free eosinophil granules (FEGs) [23]. Cytolysis is often accompanied by the formation of eosinophil extracellular traps (EETs) [21]. EETs are extracellular fiber networks composed of DNA strands decorated with granule proteins such as MBP and ECP [18]. These DNA-based traps can also incorporate intact granules (FEGs) [16] and are sometimes associated with distinctive eosinophil sombrero vesicles (EoSVs) [23], which are large tubular vesicles derived from eosinophils. Additionally, recent studies have shown that these vesicles allow for the selective release of the proteins and, perhaps, direct contact with nearby immune cells [20, 21, 24].

Interestingly, eosinophils are capable of releasing EETs even without immediate cell lysis, through a process termed EET cell death or EETosis [25]. In recent years, EETosis has garnered growing attention [21] as it has been documented in various inflammatory diseases [25] and appears to play a critical role in the pathophysiology of severe asthma [26]. External stimuli (e.g., certain cytokines or pathogens) can trigger the release of EETs, and the extent of trap formation tends to increase with prolonged exposure to these stimuli [21]. However, the molecular mechanisms regulating EETosis remain poorly understood [21].

One hallmark of eosinophil-rich inflammation involving cytolytic degranulation is the presence of Charcot–Leyden crystals (CLCs) composed of the eosinophil-derived protein galectin-10. These crystals often accompany eosinophilic inflammation (for instance, in asthma and allergic rhinitis) and serve as a biomarker of eosinophil involvement in disease [25, 27]. In affected tissues, ultrastructural analyses have shown EoSVs located near or intermingled with the extracellular decondensed chromatin that is expelled during EETosis, typically in areas containing FEGs and occasionally CLCs [23]. The presence of these vesiculotubular EoSV structures entwined with extruded DNA is considered an ultrastructural hallmark of late-stage EETosis [25]. EoSVs are thought to act as critical intermediaries facilitating granule cargo release in this process. Consistent with this idea, the number of EoSVs increases when eosinophils are exposed to inflammatory stimuli in vitro and in vivo [23]. Extracellular EoSVs are readily observed in tissues undergoing eosinophilic cytolytic inflammation; however, their precise clinical significance in eosinophil-associated diseases remains unclear [23]. Nevertheless, bulk mediator release via degranulation does not encompass the entire phenomenon. Additionally, a variety of cytokines (such as IL-4, IL-13, and TGF-β), chemokines (such as CCL11/eotaxin), lipid mediators (such as leukotrienes and prostaglandins), and growth factors (such as vascular endothelial growth factor) may be released by eosinophils in a precise manner [28].

Cytokines in the tissue microenvironment can markedly influence eosinophil degranulation, affecting both the mode of degranulation and the amount of granule content released [29]. Indeed, the nature and magnitude of eosinophil degranulation vary depending on the specific cytokine signals the cell receives [30]. For example, the pro-inflammatory cytokine tumor necrosis factor-α (TNF-α) induces oxidative stress and plasma membrane destabilization in eosinophils, thereby favoring the cytolytic mode of degranulation [17]. In contrast, it has been suggested that eosinophils employ different degranulation strategies to suit different functional needs; for instance, during piecemeal degranulation, the cells selectively release certain granule-stored components while retaining others, enabling a finely tuned response [31]. The Th1 cytokine interferon-γ (IFN-γ) generally skews eosinophils toward a more restrained degranulation profile and can even suppress eosinophil degranulation in allergic inflammatory settings [30, 32]. Notably, IFN-γ activation of human eosinophils triggers the rapid mobilization of the pre-formed chemokine RANTES (CCL5) from granules to the cell surface without causing the release of the toxic cationic proteins [33]. This exemplifies a form of regulated exocytosis mediated by SNARE proteins: vesicle-associated SNAREs on the granules engage target-membrane SNAREs on the plasma membrane (SNARE, soluble N-ethylmaleimide–sensitive factor attachment protein receptor) to form a docking complex that drives membrane fusion and selective cargo release [34].

## Dynamics of eosinophil movement

Under normal conditions, eosinophils migrate from the bone marrow to specific organs, primarily the gastrointestinal tract. Most eosinophils are found in the intestine (specifically in the segments beyond the esophagus). Other organs that normally harbor eosinophils include the uterus and mammary glands of young women, the thymus, adipose tissue (body fat), and the lungs. These locations represent the homeostatic distribution of eosinophils in healthy tissues.

Historically, eosinophils were mainly associated with responses to parasitic worm (helminth) infections and with allergic diseases. However, it is now recognized that their functions vary widely depending on the tissue environment. Studies in mice have shown that, under stable (non-inflammatory) conditions, eosinophils can play important homeostatic roles in different tissues. For example, in steady-state conditions, eosinophils contribute to the following tissue functions: Intestine: They support immunoglobulin A (IgA) antibody responses and stimulate mucus production [35]. Mammary glands: They appear to aid in normal mammary gland development [36]. Adipose tissue: They help regulate insulin sensitivity and promotes the “browning” (conversion to thermogenic brown fat) of white adipose tissue [35]. In this way, eosinophils contribute to normal physiological processes in these organs beyond their traditional role in pathogen defense.

Because eosinophils primarily reside in tissues, they normally make up less than 5% of circulating leukocytes in the blood [35] (Fig. 1). In vivo studies have shown that eosinophils remain in the bloodstream only briefly—on the order of 8–10 h on average (with a range of about 3 to 24 h) before migrating out of circulation [37, 38]. In contrast, once eosinophils enter tissues, they can persist much longer. Their half-life is roughly 36 hours in the lung and up to 5–6 days in the intestine, thymus, and uterus under steady-state conditions [37].

The extended survival of eosinophils in tissues appears to be related to tissue-specific cellular programs and local survival cues. Notably, CD11c expression has been reported on murine tissue-resident eosinophils in the intestinal lamina propria, thymus, and uterus, whereas circulating eosinophils and lung eosinophils generally show low or absent CD11c expression under steady-state conditions [39, 40]. In intestinal eosinophils, surface CD11c expression appears to be dynamically regulated and may reflect tissue adaptation or local eosinophil plasticity rather than serving as a universal marker of resident eosinophils [40, 41]. Whether CD11c directly contributes to eosinophil survival or instead marks tissue conditioning remains unresolved. In addition, eosinophil longevity is supported by the cytokine IL-5, which can inhibit eosinophil apoptosis (programmed cell death) and thereby prolong the cells’ survival in tissues [37, 42].

## Orchestration of eosinophil activity

The recruitment of eosinophils from the blood into tissues is primarily driven by chemoattractant molecules of the eotaxin family. Chief among these is eotaxin-1, also known as CCL11, a chemokine produced by epithelial cells, endothelial cells, fibroblasts, and monocytes in response to type 2 inflammatory signals such as IL-4, IL-13, and TNF-α [43]. The three eotaxins – CCL11 (eotaxin-1), CCL24 (eotaxin-2), and CCL26 (eotaxin-3) – have overlapping but distinct roles over the course of an immune response. CCL11 is the most potent chemoattractant for eosinophils [44]. It likely acts early in an inflammatory response to rapidly recruit eosinophils, whereas CCL24 and CCL26 may help sustain eosinophil accumulation and inflammation over longer periods. CCL11’s strong affinity for the receptor CCR3 on eosinophils triggers efficient signaling that leads to rapid and robust eosinophil migration into tissues [45]. The importance of CCR3 is highlighted by studies showing that loss of CCR3 – the principal receptor for eotaxin-1 [46]—results in defective homing of eosinophils to certain tissues (particularly the intestine), while eosinophil numbers in the lung and thymus remain normal in the absence of CCR3 [47]. This finding suggests that eotaxin-1 may also act through alternate receptors such as CCR5 to mediate eosinophil recruitment in some contexts [48].

In addition to chemokines like eotaxins, certain interleukins contribute to eosinophil trafficking under normal, non-diseased conditions. IL-5 and IL-13, secreted by Th2-polarized T lymphocytes and type 2 innate lymphoid cells (ILC2) [9, 49], can promote eosinophil migration into tissues even in the absence of overt inflammation [50]. However, their chemotactic influence is considerably weaker than that of eotaxin-1. IL-13 mainly serves to enhance the production of CCL26 by local tissue cells [49], while IL-5 primarily acts on eosinophil precursors and mature eosinophils: it stimulates the bone marrow production of eosinophils, increases their sensitivity to eotaxin-1, and helps maintain their survival once they enter tissues [9].

IL-5 is the cytokine most specific to the eosinophil lineage and is essential for normal eosinophil development in the bone marrow. Accordingly, the presence of IL-5 in local tissues is crucial to varying degrees for sustaining rEos populations in different organs. For example, eosinophil homing to tissues under steady-state conditions is independent of IL-5 in the lungs, partially IL-5–dependent in the gastrointestinal tract and uterus, and entirely IL-5–dependent in adipose tissue [50, 51]. In other words, local IL-5 production is absolutely required to maintain eosinophils in fat tissue, whereas other factors can compensate for the absence of IL-5 in the lungs.

In allergic and other reactive inflammatory conditions, eosinophils themselves become important sources of cytokines and chemokines that amplify type 2 inflammation. Eosinophils have been identified as significant producers of IL-5, IL-13, IL-25, and CCL26 during allergic responses, thereby contributing to a strongly Th2-skewed immune environment and subsequent eosinophil-rich inflammation [52]. Of the eotaxin family members, CCL26 actually displays the weakest chemotactic activity for eosinophils, despite also binding to CCR3. It has a lower binding affinity for CCR3 and induces only a relatively modest chemotactic response compared to CCL11 or CCL24. Notably, CCL26 is upregulated by IL-13 and is predominantly expressed by airway epithelial cells during allergic inflammation. Although its role in eosinophil recruitment is more limited, CCL26 is thought to contribute to the pathology of asthma [53]. In the allergic lung, local IL-4 and IL-13 further facilitate eosinophil infiltration by acting on the endothelium and epithelium: these cytokines increase the adhesiveness of endothelial cells by upregulating the adhesion molecule VCAM-1 [54], and they stimulate bronchial epithelial cells to secrete CCL11 [44]. The combined effect is to promote greater eosinophil adherence to blood vessel walls and subsequent migration of eosinophils into the lung tissue.

Eosinophil survival in tissues during inflammation is also prolonged by cytokines. IL-5, IL-3, and GM-CSF (granulocyte-macrophage colony-stimulating factor) act as crucial hematopoietic signals that regulate eosinophil development, maturation, and longevity [10]. IL-3 primarily drives the early expansion of eosinophil progenitor cells in the bone marrow, while IL-5 is essential for the terminal differentiation of these progenitors into mature eosinophils [49]. GM-CSF supports the maturation of eosinophil precursors and enhances the survival of both developing and mature eosinophils. Mechanistically, IL-3 exerts its effects via the IL-3 receptor (IL-3R), which consists of a specific α subunit (IL-3Rα) paired with a β-common (βc) chain that is shared with the receptors for IL-5 and GM-CSF [55]. Engagement of IL-3R by IL-3 triggers multiple intracellular signaling pathways—including JAK/STAT, MAPK, and PI3K – which act in synergy with IL-5 and GM-CSF signaling to promote eosinophil growth and resist apoptosis [56]. When IL-3 or GM-CSF signaling is dysregulated, it can lead to eosinophilic disorders characterized by excessive eosinophil accumulation, tissue damage, and chronic inflammation [55, 57].

Beyond their role in inflammation, eosinophils actively participate in regulating immune responses. Eosinophils promote humoral immunity by priming B cells, which can enhance antibody production [35]. They also play a central role in shaping type 2 immunity: eosinophils can function as antigen-presenting cells to activate CD4^+^ T cells, and they secrete an array of type 2 cytokines from their granules—notably IL-4, IL-5, and IL-13 – which influence the immune environment [35]. Through these actions, eosinophils help orchestrate the balance between Th1 and Th2 immune responses, closely regulating the shift toward Th2 immunity when appropriate [58].

## Molecular triggers of eosinophil function

Eosinophils are terminally differentiated granulocytes that develop in the bone marrow and circulate briefly in the bloodstream before migrating into tissues. These cells are characterized by their bilobed nucleus and large cytoplasmic granules filled with cytotoxic proteins and enzymes. In parasitic infections, eosinophils degranulate and release potent molecules such as major basic protein and eosinophil peroxidase that directly damage helminths and other parasites. However, in allergic airway diseases like atopic asthma, the same degranulation process can injure host tissues. Elevated eosinophil counts in the bronchial mucosa of asthmatic patients correlate with epithelial damage and disease severity [59]. This cytotoxic function is complemented by the ability of activated eosinophils to extrude mitochondrial DNA and granule proteins into the extracellular space, forming EETs. These net-like structures immobilize pathogens but can also contribute to tissue injury during chronic inflammation [60]. EET formation requires strong activation signals: priming with interferon-γ and high concentrations of IL-5 increases the propensity of eosinophils to release DNA nets. Experimental studies show that both IL-5 and thymic stromal lymphopoietin (TSLP) can directly induce EET formation [61], and IL-5 priming enhances EET release in severe asthma [62]. The molecular pathways underlying EETosis are still incompletely understood and remain an important area for future research [26].

Beyond their cytotoxic role, eosinophils serve as immunomodulatory cells. They synthesize and secrete a surprisingly broad spectrum of cytokines, chemokines, and lipid mediators [63]. Upon activation, eosinophils release pro-inflammatory cytokines such as IL-2, IL-4, IL-5, IL-10, IL-12, IL-13, IL-16, IL-18, and transforming growth factor-α/β, chemokines including RANTES and eotaxin-1, and lipid mediators like platelet-activating factor and leukotriene C4 [64]. These mediators regulate adhesion molecule expression, cell trafficking, vascular permeability, mucus secretion, and smooth-muscle contractility, thereby amplifying type 2 inflammation and airway hyper-responsiveness. Eosinophils can also initiate antigen-specific immune responses by functioning as antigen-presenting cells. Under appropriate activation conditions, eosinophils express major histocompatibility complex class II molecules and co-stimulatory proteins such as CD86 and CD30 ligand [65]. This allows them to present antigenic peptides to T lymphocytes and modulate adaptive immunity, illustrating their role as a bridge between innate and adaptive responses [66].

Epithelial-derived cytokines are critical regulators of eosinophil activation and survival. The “alarmins” TSLP, IL-25 and IL-33 are released by damaged airway epithelium and stimulate type 2 innate lymphoid cells to produce IL-5, thereby promoting eosinophil differentiation, recruitment, and survival [67]. TSLP and IL-33 can act directly on eosinophils by engaging the TSLP receptor and ST2 (the IL-33 receptor), inhibiting apoptosis and extending eosinophil lifespan [68]. These signals not only amplify Th2 responses but also enhance eosinophil function by increasing cytokine secretion and responsiveness to chemokines.

## Revisiting eosinophil diversity

In recent years, multiple studies have proposed that eosinophils comprise distinct subtypes. A central unresolved question is whether these states reflect a single lineage at different activation or maturation stages, or, by analogy with Th1 vs. Th2 lymphocytes, represent intrinsically distinct populations generated in the bone marrow with stable functional programs. A pivotal murine study by Mesnil et al. [68] addressed this issue in an asthma model, delineating lung resident, homeostatic/physiological eosinophils (rEos) versus allergen-recruited inflammatory eosinophils (iEos). This framework is influential but should be interpreted alongside evidence that eosinophil phenotypes and functions differ substantially across tissues. Across complementary experiments in lung and blood, the investigators demonstrated clear, reproducible differences between eosinophil populations in allergic asthmatic mice and healthy controls. In mice, the rEos–iEos distinction is supported by nuclear morphology, membrane protein expression, and tissue localization. rEos display canonical eosinophil features, including red-staining granules rich in MBP and EPO—and co-express CCR3, Siglec-F, and CD125 (the IL-5 receptor α subunit) [35, 42, 63]. Depending on the tissue, rEos can also express CD11b (intestine, thymus, adipose tissue), F4/80 (mammary gland, lung, adipose tissue), and activation/adhesion molecules such as CD69 and CD44 (intestine, thymus) [42, 63, 68]. Most tissue rEos possess a segmented nucleus and are CD11c positive [12, 62].

The lung is a notable exception. Lung rEos resemble resting blood eosinophils, exhibiting a ring-shaped nucleus, CD62L expression, intermediate Siglec-F, and CD11c negativity [68, 69]. In mice, a ring-shaped nucleus often indicates cellular immaturity [65], suggesting that lung rEos retain an immature phenotype after dissemination to the airways. Despite this, they undergo gradual degranulation and retain phagocytic capacity, indicating preserved effector functions.

**Fig. 2:**
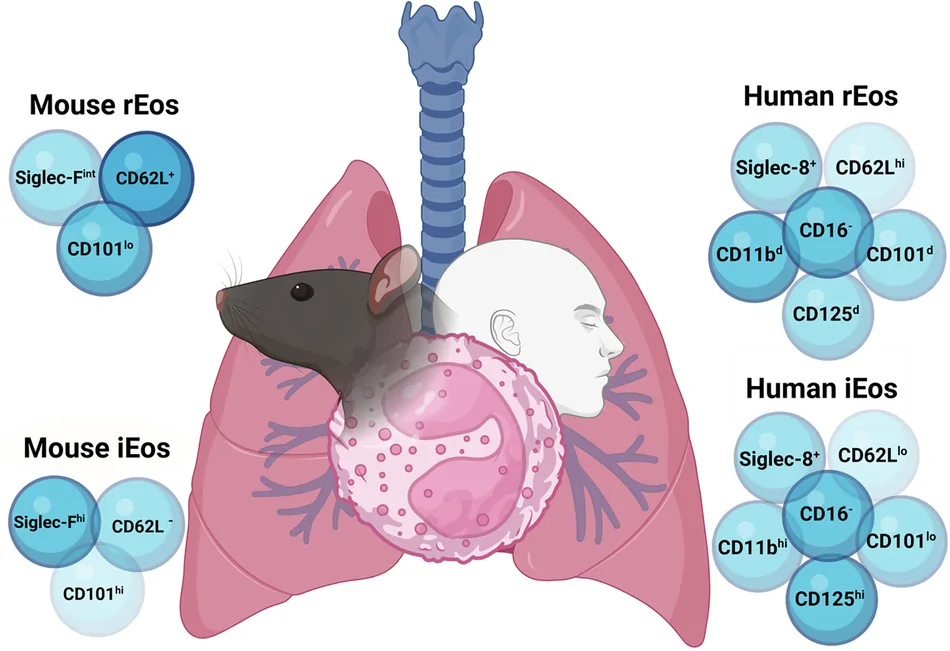
Phenotyping of blood eosinophil subtypes in mouse and human. These subsets reflect homeostatic versus inflammatory functions across species—rEos: resident eosinophils, iEos: inflammatory eosinophils.

Importantly, during allergic airway inflammation, the number, localization, and morphological/phenotypic/transcriptomic characteristics of lung rEos remain stable and distinct from iEos. By contrast, iEos are robustly recruited to the lungs upon allergen exposure and are defined as SiglecF^hi^ CD62L^-ve^CD101^hi^ cells with a segmented nucleus; CD101 functions as an iEos marker and is not expressed on lung rEos. These observations support a true rEos-iEos dichotomy and further suggest that analogous subsets exist in the circulation of asthmatic mice, implying that differentiation may occur prior to tissue recruitment. Consistent with this cross-species framework, Mesnil et al. [68] also compared human samples, showing that parenchymal rEos in non-asthmatic lungs (Siglec-8^+^ CD62L^hi^ IL-3R^lo^) are phenotypically distinct from iEos recovered from asthmatic sputum (Siglec-8^+^ CD62L^lo^ IL-3R^hi^), thereby confirming key murine findings in humans (Fig. 2).

However, eosinophil heterogeneity is not limited to the lung, and the rEos/iEos framework may not capture all tissue-specific eosinophil states. Recent studies have identified distinct transcriptional and functional programs in gastrointestinal, adipose-resident, and thymic eosinophils, including populations associated with mucosal immunity, host defense during colitis, metabolic regulation, and thymocyte maturation [70–73]. These data suggest that eosinophil states may reflect both tissue imprinting and activation history and, in some settings, may follow a continuum from basal or homeostatic to activated phenotypes rather than a strictly dichotomous resident-versus-inflammatory classification [71, 74–76]. Importantly, several tissue-associated eosinophil populations have been implicated in host-protective or immunoregulatory, underscoring that eosinophil activation should not be equated automatically with pathogenicity [71–73].

Subsequent studies in horses [77] and humans [78–82] have extended the rEos-iEos framework, particularly in airway disease, although marker combinations, tissue context, and sample-processing methods vary across studies (Table 1). Matucci et al. [78] examined eosinophil subpopulations in peripheral blood and nasal polyps from patients with severe eosinophilic asthma (SEA) with chronic rhinosinusitis with nasal polyps (CRSwNP), alongside non-severe asthma (NSEA), allergic rhinitis without asthma, and healthy volunteers. They also profiled eotaxin-3 and eotaxin-1 in nasal polyps. SEA patients showed increased peripheral blood eosinophils (Siglec8^+^ CD45^+^ CD16^–^) and two subtypes stratified by CD62L. The CD62L^lo^ subset—enriched in SEA—displayed high CCR3 and CD69 with low CD125, CRTH2, CD86, CD28, CD101, and VLA-4. Nasal polyps contained a higher proportion of CD62L^lo^ eosinophils than blood, and within polyps, IL-3R, IL-5R, CD69, and CD86 were upregulated on CD62L^lo^ cells relative to their blood counterparts. Notably, eotaxin-3 expression correlated with the percentage of CD62L^lo^ eosinophils in polyps. In line with Mesnil et al. [68], CD62L^lo^ aligns with iEos and CD62L^bright^ with rEos [68, 83].

**Table 1:** Percentages of rEos and iEos in human peripheral blood.

Patient Group	Matucci et al. (2023)—*SEA vs non-SEA (110)*	Vultaggio et al. (2023)—*SEA vs non-SEA (111)*	Cabrera López et al. (2023)—*Asthma vs COPD (112)*	Mycroft et al. (2023)—*Mild Asthma vs COPD (115)*	Januskevicius et al. (2019) —*AA vs SNEA (116)*
**Samples**	SEA: *n* = 23, NSEA: *n* = 15, control: *n* = 15	SEA: *n* = 112, NSEA: *n* = 19	Asthmatics: *n* = 10, COPD: *n* = 10, healthy control: *n* = 10	Mild asthmatics: *n* = 14, Mild COPD: *n* = 15, controls: *n* = 11, median: IQR	AA: *n* = 10, SNEA: *n* = 16, control: *n* = 11
**Non-severe Asthma (NSEA)**	205± * cells/µL; ≈3.3% iEos	700 ± 63 cells/µL; 8.5% ± 0.7%	380 ± 164 cells/µL; 21.5% ± 16.6%	219 (137–390) cells/µL; ~5% iEos	530 ± 80 cells/µL; ≈62.8% iEos
**Severe Eosinophilic Asthma (SEA)**	604 ± 79 cells/µL; 12.3% ± 3.5% iEos	932 ± 100 cells/µL; 10.5% ± 1.1%	730 ± 91 cells/µL; 30.0% ± 13.1% iEos	*N/A* (group not studied)	680 ± 110 cells/µL; 36.2% ± 3.8% iEos
**COPD Patients**	*N/A* (group not studied)	*N/A* (group not studied)	239 ± 181 cells/µL; 0.7% ± 1.1% iEos	175 (127–282) cells/µL; ~2% iEos	*N/A* (group not studied)
**Healthy Controls**	109 ± 22 cells/µL; 2.25% ± 1.5% iEos	*N/A* (group not studied)	203 ± 92 cells/µL; 0.67% ± 1.72% iEos	171 (55–185) cells/µL; ~2% iEos	170 ± 20 cells/µL; 48.7% ± 5.9% iEos
**Phenotyping**	Flow cytometry: iEos: CD62L^low^; rEos CD62L^bright^	Flow cytometry: iEos: CD62L^low^; rEos: CD62L^bright^	Siglec-8/CD62L: iEos = CD62L^low^ IL-3R^hi^; rEos = CD62L^+^ IL-3R^lo^	Flow cytometry: iEos = CD62L^low^ CD11b^+^	Flow cytometry & Magnetic separation by CD62L: iEos = CD62L^low^

This table has been modified and extended from Sánchez Santos A et al. [119]; IQR: Interquartile Range.

Vultaggio et al. [79] extended these observations to clinical outcomes. In a cohort of 112 patients (51 naive; 61 previously biologic-treated), they evaluated iEos (CD62L^lo^) in blood and assessed modulation by mepolizumab. Nineteen naive patients were analyzed longitudinally pre-/post treatment (100 mg SC mepolizumab/4 weeks), and 23 patients already receiving mepolizumab were profiled at study entry; in vitro assays tested the effects of IL-5 and anti-IL-5 on CD62L expression. The proportion of CD62L^lo^ cells correlated with patient-reported outcomes, including ACT and SNOT-22 scores, and with exacerbations in untreated individuals. In the naive group, mepolizumab reduced CD62L^lo^ frequencies while increasing the CD62L^bright^ fraction, paralleling the distribution seen in healthy controls and associating with improved disease control. Multiple cytokines (IL-3, GM-CSF, IL-33, TSLP, TNF-α) modulated CD62L expression, whereas IL-4 did not, underscoring a nuanced, cytokine-dependent regulation of the iEos phenotype.

Fricker et al. [82] similarly profiled eosinophil subpopulations in severe asthma, corroborating findings from Matucci et al. [78] and Cabrera López et al. [80]. In a longitudinal subset (*n* = 30) sampled 4–24 weeks after initiating mepolizumab (*n* = 20) or benralizumab (*n* = 10), both biologics depleted iEos to a comparable extent. However, mepolizumab preferentially spared a residual rEos population, whereas benralizumab depleted both rEos and iEos. These data reinforce that an elevated proportion of circulating iEos is associated with poorer asthma control [82] and that the biologic mechanism of action differentially sculpts the eosinophil landscape.

Cabrera López et al. [80] emphasized the prevalence of iEos in asthma—typically greater than 20% of total eosinophils—compared to minimal percentages (<1%) in healthy subjects, smokers without COPD, and COPD patients. In COPD, iEos proportions appeared independent of disease severity, treatment, and exacerbation history. Importantly, the iEos fraction is uncoupled from the total eosinophil count: some patients with only ~250 eosinophils/μL nonetheless exhibited up to 45% iEos, potentially resolving discrepancies between blood counts and tissue eosinophilia. Methodologically, Cabrera López et al. [80] analyzed fresh, unfractionated whole blood (100 μL) from 10 stable subjects across four groups (COPD, asthma, smokers without COPD, healthy volunteers) and validated observations in 59 COPD and 17 asthma patients. Phenotyping was performed according to Mesnil’s criteria, incorporating additional markers such as CD125 and CD11b (Table 2). iEos were identified as CD45^+^ Siglec8^+^ CD16^–^ CD62L^lo^ CD11b^hi^ CD125^hi^ CD101^lo^, and rEos as CD45^+^ Siglec8^+^ CD16^–^ CD62L^hi^ CD11b^d^ CD125^d^ CD101^d^, using flow cytometry and confocal microscopy. Mycroft et al. [83] analyzed eosinophil phenotypes in blood and sputum of COPD, asthma, and control subjects. They found higher expression of CD125, CD193, CD14, and CD62L in blood than in sputum eosinophils across groups, with increased CD193^+^ and CD66b^+^ sputum eosinophils in COPD and more CD11b^+^ eosinophils in asthma. These findings suggest a distinct eosinophil phenotype in COPD compared to asthma or healthy controls. In line with Cabrera López et al. [80], iEos were prevalent in asthma (>20%) but minimal in COPD and healthy subjects (<1%), highlighting that eosinophil phenotyping may provide more insight than absolute counts.

**Table 2:** Comparative expression of iEos markers in human blood, nasal polyp, and lung.

Marker	Blood	Nasal Polyps iEos	Lung iEos	Notes/Relevance
**CD62L (L-selectin)**	Low	Low	Low	Reduced expression reflects tissue homing vs. naïve circulating eosinophils
**CD125 (IL-5Rα)**	High	High	Variable (moderate–high)	Key receptor for IL-5–driven survival/activation
**CD11b (Integrin αM)**	High	High	High	Adhesion & migration; upregulated in activated tissue eosinophils
**CD101**	Low	High	High (variable in asthma)	Activation marker; linked to tissue remodeling
**CCR3 (Eotaxin receptor)**	High	High	High	Crucial for chemotaxis; consistent across compartments
**Siglec-8**	High	High	High	Inhibitory receptor; therapeutic target (e.g., lirentelimab)
**CD69**	Low	High	High	Early activation/tissue residency marker
**PD-L1 (CD274)**	Low	Variable ( ↑ in chronic rhinosinusitis)	High (in severe asthma)	Immune regulation via T-cell suppression
**HLA-DR**	Rare/Low	Variable	Increased in the inflamed lung	Suggests antigen-presentation potential in subsets
**CD44 / CD45RO**	Moderate	High	High	Memory/activation phenotype in tissue eosinophils
**CD38**	Low	High	High	Associated with metabolic activation and airway remodeling
**CD123**	Low	High	High	Critical for eosinophil differentiation, survival, and activation

For this review, asthmatic cohorts were categorized into SEA and NSEA. Divergent sample-processing workflows likely account for some variability in reported iEos/rEos proportions across studies. For example, Januskevicius et al. [84] reported higher iEos in SEA; this may reflect magnetic selection based on CD62L, which could downregulate CD62L upon bead engagement, artificially enriching the CD62L^lo^ gate (Table 1). By contrast, the proportions described by Matucci et al. [78], Vultaggio et al. [79], and Cabrera López et al. [80] are more concordant, yet residual differences remain that cannot be ascribed to gating algorithms alone. One plausible explanation is cell loss attributable to preparation methods: Ficoll density gradients [78, 79] may impose greater mechanical and osmotic stress than erythrocyte lysis [80], potentially leading to disproportionately reduced recovery of fragile subsets—particularly iEos. Finally, geographic and environmental factors may modulate baseline eosinophil biology. For instance, the Canary Islands are noted for higher peripheral eosinophil numbers, which could contribute to the distinct iEos distributions reported for SEA and NSEA cohorts relative to other settings. Together, these data strengthen the concept that rEos and iEos are phenotypically and functionally distinct, with differences evident in blood and tissues, regulated by local cytokine milieus and therapeutic interventions, and sensitive to pre-analytical variables that must be harmonized across studies (Tables 1, 2).

## Divergent functions of eosinophil subsets in airway disease

The concept of iEos and rEos is relatively new. Evidence regarding their mechanisms and roles across diseases remains limited, and key clinical contexts are still unexplored. To date, no studies have specifically evaluated these phenotypes during exacerbations, nor in patients treated with monoclonal antibodies other than mepolizumab and benralizumab. Moreover, their functional roles have not yet been published and thus remain speculative. This evidence gap has been explicitly highlighted by the EAACI task force on new molecular insights and clinical functions of eosinophils [85], which calls for focused research on this topic in the coming years.

Even with these limitations, several hypotheses emerge regarding potential clinical utility. One is that a threshold proportion of iEos, potentially in the 8–10% range, could suffice to classify an asthmatic patient as Th2-high. Approximately 20% of patients with severe asthma show discordance between blood and tissue eosinophils; different eosinophil subpopulations may explain this discrepancy. As discussed above, some asthmatic patients have low total blood eosinophils yet a high fraction of iEos; this may correspond to the iEos described in non-eosinophilic asthma by Fricker et al. [82]. We speculate that iEos, by virtue of their molecular surface markers, are preferentially recruited to sites of inflammation. Thus, blood iEos might serve as a surrogate of Th2 signaling even when the total eosinophil count does not exceed 250 cells/mm³. If validated, iEos frequency could enable endotyping in severe asthma and help identify candidates for anti-IL-5/IL-5R therapy—even in patients without overt peripheral eosinophilia. This reasoning may also contribute to understanding why Tezepelumab demonstrates efficacy in some non-Th2 asthma phenotypes; however, such benefit likely reflects only part of the explanation, given Tezepelumab’s broader impact on multiple cell types and pathways beyond IL-5 inhibition. Consistent with this, data from Vultaggio et al. [79] suggest that iEos proportions may correlate more closely with symptom control (asthma and nasal polyposis) than total blood eosinophils and could potentially serve as a biomarker of control in patients treated with mepolizumab.

In COPD, results with biological therapies have generally been disappointing. At present, dupilumab appears to reduce exacerbations, with the greatest benefit observed in patients with high blood eosinophils, high FeNO, and elevated IgE. Precise patient selection is therefore critical. Eosinophil subpopulations may assist in identifying those most likely to respond to anti-eosinophilic treatments. Notably, Cabrera López et al. [80] reported that COPD patients—including those with elevated blood eosinophils, harbored <1% iEos. Detecting COPD patients with a meaningful iEos fraction could thus be highly informative for therapeutic stratification and selection of candidates for monoclonal antibody therapy.

**Fig. 3:**
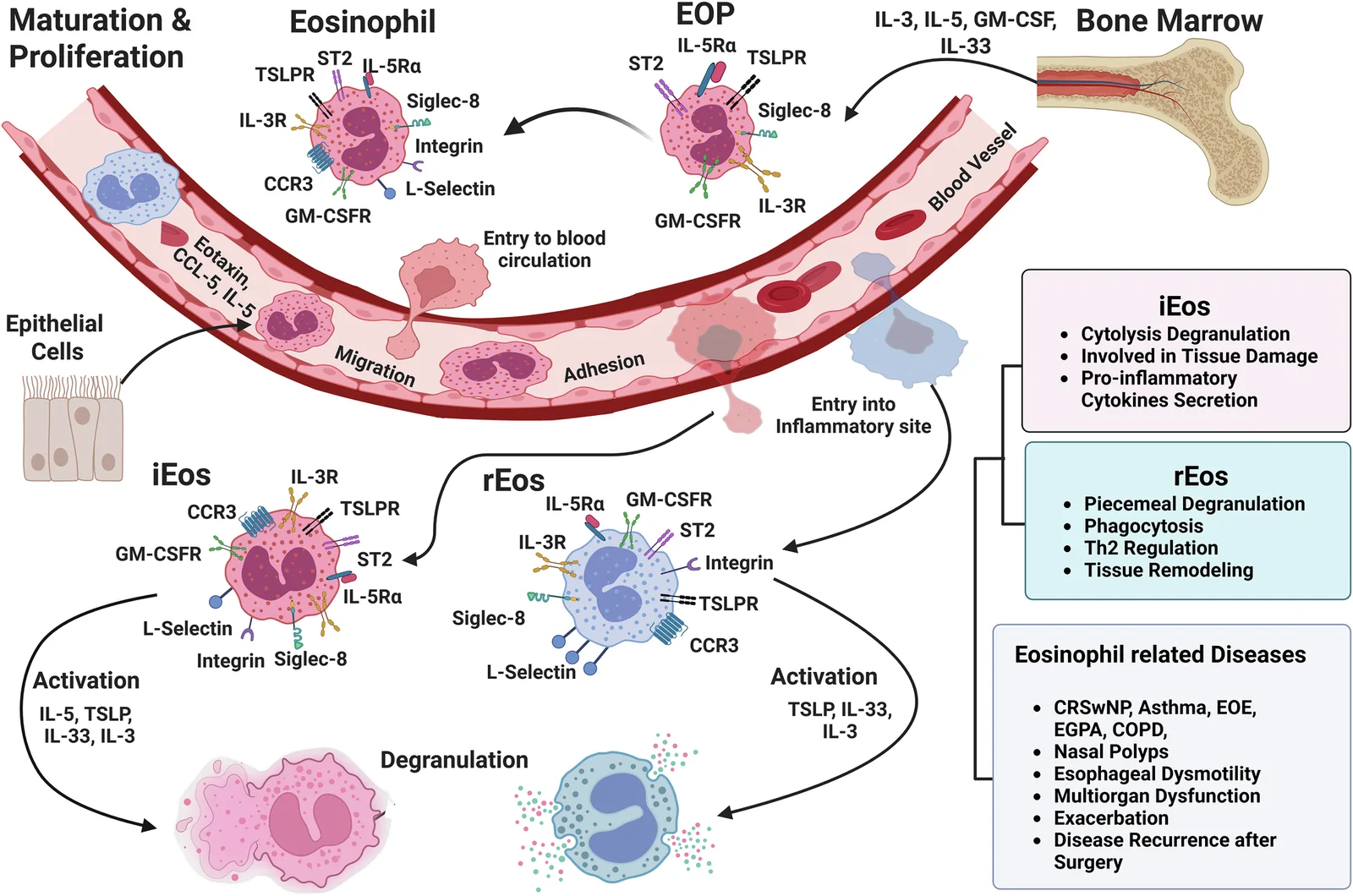
The different roles of eosinophil subgroups in chronic inflammatory diseases. Elevated levels of pro-inflammatory cytokines in inflamed tissues drive the proliferation and differentiation of eosinophil progenitors (EoP) into distinct resident (rEos) and inflammatory (iEos) eosinophil subpopulations before they enter the bloodstream. Chemokines recruit these eosinophils to sites of inflammation, where upregulated adhesion molecules on the vascular endothelium slow their transit and facilitate tissue infiltration. Within this microenvironment, eosinophils become activated and release cytokines and granule proteins. These mediators exacerbate inflammation and tissue damage, contributing to the pathogenesis of chronic inflammatory diseases such as CRSwNP, asthma, EoE, and EGPA. CRSwNP: chronic rhinosinusitis with nasal polyps, EGPA: eosinophilic granulomatosis with polyangiitis, EoE: eosinophilic esophagitis, EoP: eosinophil progenitors, iEos: inflammatory eosinophil, rEos: resident eosinophil.

A further knowledge gap concerns temporal dynamics: whether iEos:rEos proportions shift between stable disease and exacerbations. It is plausible that iEos increase during exacerbations in both asthma and COPD, which, if confirmed, would have direct implications for biologic candidacy and treatment timing. Addressing this will require prospective, longitudinal studies that sample at baseline, during exacerbation, and in convalescence; use standardized pre-analytical workflows (to minimize artifactual modulation of markers such as CD62L); and apply harmonized multiparameter flow cytometry panels that include the defining markers for iEos/rEos. Collectively, and pending such validation, current data support the possibility that iEos and rEos represent clinically informative eosinophil states whose measurement may eventually augment conventional biomarkers for endotyping, prognostication, and therapeutic selection in airway disease [79, 80, 82, 85] (Fig. 3).

## Harnessing eosinophils for anti-tumor strategies

**Fig. 4:**
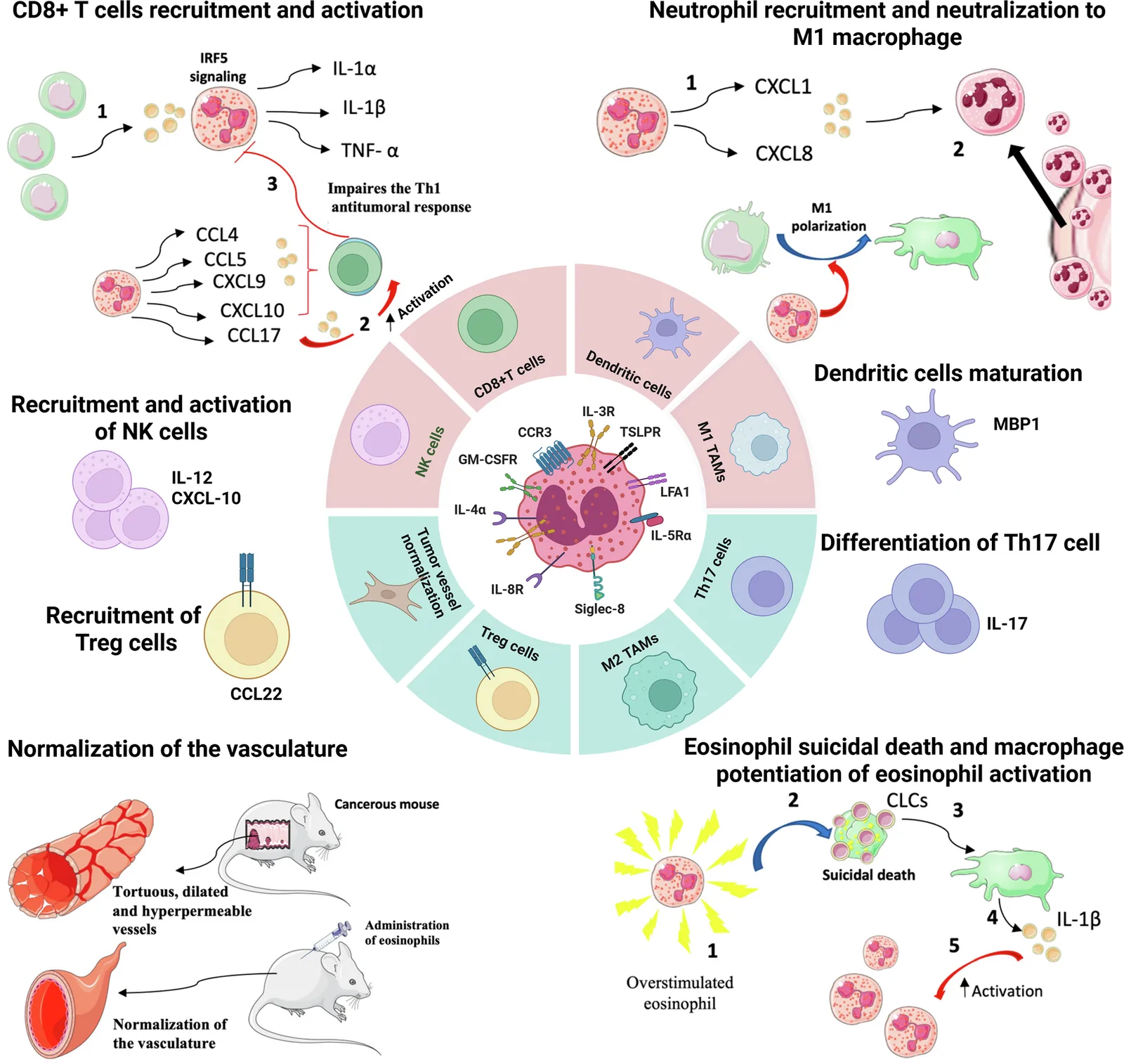
Eosinophil recruitment and anti-tumor functions. Tumor-derived cytokines and alarmins (e.g., IL-5, IL-33) recruit and activate eosinophils at tumor sites. Activated eosinophils exert direct cytotoxic effects through degranulation (MBP-1, RNases) and indirect effects by recruiting NK cells, CD8⁺ T cells, and dendritic cells, and by polarizing macrophages toward an M1 phenotype. They also modulate tumor vasculature, increasing leakiness and promoting necrosis. Abbreviations: IL, interleukin; CCL, CC-chemokine ligand; CXCL, CXC-chemokine ligand; TNF-α, tumor necrosis factor-α; CCR, CC-chemokine receptor; TSLPR, thymic stromal lymphopoietin receptor; LFA-1, leukocyte function-associated antigen-1; GM-CSFR, granulocyte–macrophage colony-stimulating factor receptor.

Both myeloid and lymphoid malignancies have well-documented associations with eosinophilia. In myeloid neoplasms (such as chronic myelogenous leukemia, chronic eosinophilic leukemia, and acute myeloid leukemia) [86], eosinophilia arises directly from the clonal proliferation of malignant myeloid progenitor cells. By contrast, in lymphoid neoplasms associated with eosinophilia, the eosinophil expansion is typically driven indirectly by cytokine secretion [87]. T-cell neoplasms are most frequently linked to eosinophilia (e.g., cutaneous T-cell lymphoma, adult T-cell leukemia/lymphoma, angioimmunoblastic T-cell lymphoma), although certain B-cell malignancies (notably Hodgkin lymphoma and B-lymphoblastic leukemia/lymphoma) have also been implicated [88, 89] (Fig. 4).

In practice, unexplained eosinophilia in peripheral blood or bone marrow usually prompts pathologists to search for an underlying myeloid or lymphoid neoplasm, since reactive (non-neoplastic) eosinophilia provides limited diagnostic information. Recent publications offer comprehensive reviews of this topic, including molecular-level diagnostic approaches [90]. Additionally, eosinophilia has been observed in some non-hematologic malignancies (such as breast, pancreatic, and lung cancer), where it is sometimes regarded as a paraneoplastic syndrome [91].

## Pharmacotherapy in eosinophil-driven diseases

Glucocorticoids have traditionally been the cornerstone treatment for eosinophilic diseases, as they decrease eosinophil levels and broadly reduce inflammation [92]. Corticosteroids—administered systemically or by inhalation—remain the gold-standard therapy, primarily by suppressing type 2 inflammation and reducing eosinophil-promoting mediators like IL-5 and eotaxin. However, glucocorticoids are associated with significant side effects and often prove inadequate for controlling severe disease.

In recent years, a major therapeutic advance has been the development of biologic medications that specifically target eosinophils or the pathways governing their recruitment and survival. Monoclonal antibodies against IL-5 (e.g., mepolizumab, reslizumab) or against the IL-5 receptor α chain (benralizumab) effectively reduce eosinophil levels in blood and tissues, leading to improved outcomes in many patients with severe eosinophilic asthma [93, 94].

Another promising target is Siglec-8, an inhibitory receptor expressed on eosinophils and mast cells. Lirentelimab, a monoclonal antibody targeting Siglec-8, induces eosinophil death and markedly lowers eosinophil numbers in blood and tissue [95]. Clinical evidence for Siglec-8 blockade remains disease-specific and incompletely resolved. In a phase 2 trial of eosinophilic gastritis and/or eosinophilic duodenitis, lirentelimab reduced gastrointestinal eosinophil burden and improved symptom scores [95]. In chronic spontaneous and inducible urticaria, an open-label proof-of-concept study suggested potential clinical activity, although the absence of a placebo control limits interpretation [96]. Together, these findings indicate that eosinophil depletion or modulation may have context-dependent clinical effects and underscore the need to distinguish eosinophils as pathogenic drivers, biomarkers of tissue inflammation, or participants in broader immune circuits.

Patient selection for eosinophil-targeted therapies has largely been based on peripheral blood eosinophil counts [97]. However, blood eosinophil levels have important limitations: elevated blood eosinophils are not always reliable indicators of eosinophilia, correlate with disease severity, or predict therapeutic responsiveness [98]. Indeed, even in patients with high baseline eosinophil counts and clear eosinophil reductions on therapy, clinical benefits have sometimes been only modest. For example, in eosinophilic esophagitis (EoE), treatments like benralizumab (anti-IL-5Rα) or Siglec-8 blockade have yielded limited improvement despite effectively depleting tissue eosinophils [99]. Similarly, while anti-IL-5/IL-5Rα therapies help many patients with severe eosinophilic asthma, a subset of patients fails to respond despite exhibiting a pronounced eosinophilic inflammatory signature [100].

This variability in treatment response may reflect several factors. For instance, some patients may have parallel non–type 2 inflammatory pathways driving disease [101] or genetic variants that alter type 2 cytokine signaling [102]. In other cases, disease activity might be sustained by alternative, tissue-specific eosinophil survival mechanisms [103] or by therapy-resistant eosinophil subsets residing in particular anatomical niches [104].

Importantly, eosinophil activity (such as degranulation) may be a more accurate indicator of disease severity and treatment response than eosinophil counts alone. Conditions with similar degrees of eosinophil infiltration can exhibit markedly different levels of eosinophil degranulation [19]. For example, sputum studies highlight the contribution of eosinophil degranulation products to airway disease [105], and extracellular free eosinophil granules have been proposed as indicators of an ongoing type 2 inflammatory process or a positive “biologic” treatment response [106]. Eosinophil peroxidase levels have likewise been suggested to be a more reliable biomarker of active eosinophilic inflammation than simple eosinophil counts [107].

The mechanisms underlying clinical improvement in patients who do respond to eosinophil-targeted therapies are likely multifactorial. Although IL-5/IL-5Rα–directed biologics are often labeled “anti-eosinophil” therapies, eosinophils are not the only cells expressing IL-5Rα. Basophils, certain B cells (plasmablasts), airway epithelial cells, and fibroblasts can also express IL-5Rα [108]. Likewise, Siglec-8 is present on mast cells and, to a lesser extent, on basophils [109]. Therefore, the clinical efficacy of agents like benralizumab, reslizumab, mepolizumab, or lirentelimab may partly depend on effects on these non-eosinophil cell populations—an area that warrants further investigation.

Beyond agents that directly target eosinophils, several other biologics attenuate eosinophilic inflammation indirectly by suppressing upstream type 2 immune pathways or epithelial “alarmin” signals. For example, dupilumab (which blocks IL-4Rα and thus interferes with IL-4 and IL-13 signaling) and the anti-TSLP antibody tezepelumab can reduce eosinophilic inflammation in asthma and other conditions [67, 92]. Biologics targeting IL-33 or its receptor ST2 are also in late-stage clinical development [110]. In addition, the oral investigational drug dexpramipexole has shown the ability to markedly deplete blood and tissue eosinophils, although its precise mechanism of action remains unknown [111].

**Fig. 5:**
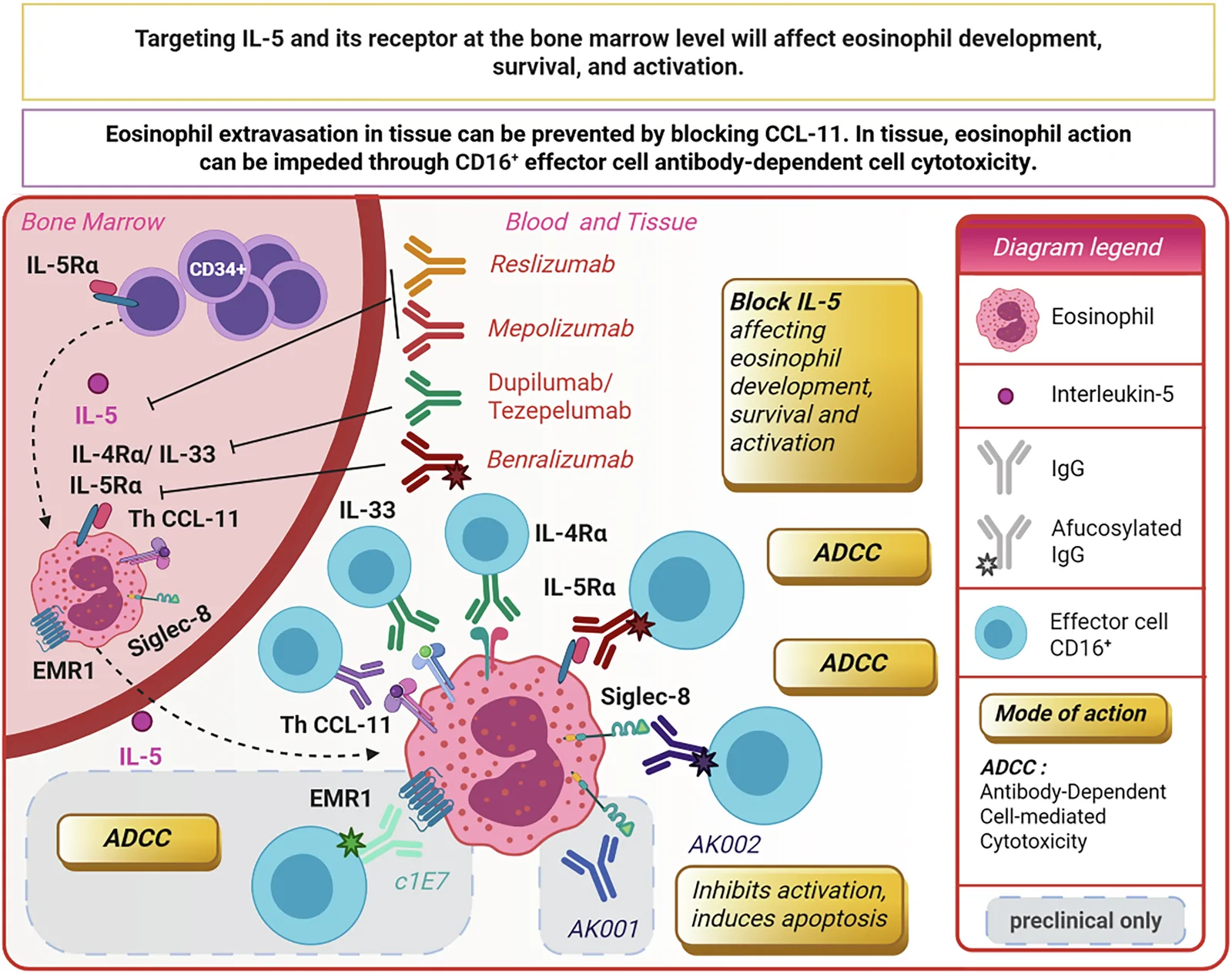
Therapeutic targets modulating eosinophil development, activation, and survival. IL-5 signaling through IL-5Rα in the bone marrow promotes eosinophil differentiation from CD34^+^ progenitors, while IL-4/IL-13 and CCL-11 (eotaxin) further support eosinophil activation and tissue recruitment. Monoclonal antibodies such as reslizumab, mepolizumab, benralizumab, and dupilumab/tezepelumab block IL-5 or its receptor, thereby reducing eosinophil survival and activation. Benralizumab additionally induces antibody-dependent cell-mediated cytotoxicity (ADCC) through CD16^+^ effector cells. Experimental antibodies (c1E7, AK002, AK001) inhibit eosinophil activation or promote apoptosis. Overall, targeting IL-5/IL-5R signaling, CCL-11, or effector cell-mediated ADCC represents key therapeutic strategies for limiting eosinophil-driven inflammation.

A persistent challenge across all eosinophil-modulating therapies is the unpredictability of individual patient responses. This uncertainty has prompted efforts to discover new biomarkers that can guide the selection and tailoring of treatments. Potential approaches include detailed transcriptomic and proteomic profiling of eosinophils from patient samples, advanced imaging techniques to assess the spatial distribution and activation state of eosinophils in affected tissues, and integrated clinical–molecular frameworks that combine biomarker data with imaging findings and traditional clinical parameters. Ultimately, a deeper understanding of eosinophil heterogeneity and function in different disease contexts will be essential for optimizing personalized treatment strategies and improving patient outcomes (Fig. 5).

## Limitations of Single-Cell Eosinophil Profiling

Single-cell RNA sequencing (scRNA-seq) has become a powerful approach to dissect immune cell heterogeneity, but eosinophils present unique technical obstacles [112]. These granulocytes are fragile and easily activated during isolation, which can introduce artifactual changes in their gene expression profiles [112]. Moreover, eosinophils are packed with ribonucleases (RNases) in their granules, which can rapidly degrade RNA if the cells become stressed or lyse during processing [113]. Consequently, eosinophils have often been underrepresented in single-cell studies because they may be lost or transcriptionally altered by the time of analysis.

To minimize these artifacts, researchers have developed gentle, rapid handling protocols for eosinophil isolation. For example, directly encapsulating freshly isolated eosinophils into microfluidic scRNA-seq platforms can reduce activation time and preserve the cells’ native transcriptomic state [114]. Specialized “stress-free” processing methods likewise aim to prevent degranulation and RNA degradation, yielding higher-quality eosinophil transcriptomes [115]. Alternatively, single-nucleus RNA sequencing (snRNA-seq) can be employed to profile eosinophils: by capturing messenger RNA from isolated nuclei, this approach bypasses the cytoplasmic RNases and can provide transcriptomic insights even when whole-cell sequencing is problematic. Using these strategies, researchers are increasingly able to obtain reliable single-cell data for eosinophil phenotyping.

Spatial transcriptomic methods profile cells in situ within intact tissues, preserving the spatial context that is lost during cell isolation [116]. This histology-based approach avoids the need to physically isolate eosinophils and thus circumvents some issues of cell fragility. Recent technological advances have made high-plex spatial transcriptomics feasible. For example, several platforms—such as 10x Genomics Visium and Xenium, NanoString GeoMx and CosMx, and Vizgen’s MERFISH-based MERSCOPE—can generate transcriptome-wide gene expression maps directly from tissue sections at near-cellular resolution [117]. These technologies enable investigators to pinpoint eosinophil-specific gene expression signatures within their native tissue microenvironments.

However, analyzing spatial transcriptomic data presents its own challenges. Delineating individual eosinophils in crowded tissue sections and handling the enormous volume of data generated requires sophisticated computational tools [117]. Recent advances in spatial statistics and transcript-to-cell assignment algorithms are improving the accuracy of cell identification and the interpretation of spatial omics data [118].

In addition, researchers are integrating spatial transcriptomics with multiplexed imaging modalities to gain a more comprehensive view of eosinophil biology. For instance, transcriptomic maps can be co-registered with immunofluorescence or mass-spectrometry-based imaging (such as MALDI or imaging mass cytometry) to correlate eosinophil gene expression with protein markers and metabolic signals in situ [116]. These integrated approaches help validate transcriptomic findings and shed light on eosinophil functional states across different tissue niches.

Together, single-cell and spatial transcriptomic tools are providing unprecedented insights into eosinophil subsets and their functions in disease. By capturing eosinophils’ gene expression either in suspension or within intact tissues, researchers can distinguish different activation states (e.g., homeostatic vs. inflammatory) and observe how local micro-environmental cues influence eosinophil behavior. Mapping eosinophils in the context of surrounding cells and signals is especially valuable for identifying which eosinophil phenotypes drive pathology versus aid in tissue repair. Ultimately, these advanced phenotyping methods will refine our understanding of eosinophil heterogeneity in conditions like asthma, EoE, and other inflammatory diseases (including certain cancers).

## Conclusion and future directions

Eosinophils are granulocytic cells that have long been regarded as purely inflammatory and defensive, primarily in the context of pathology. This view has shifted as accumulating evidence reveals homeostatic roles and cooperative interactions with other immune cells. A key advance is the recognition of phenotypically distinct eosinophil states, including, but not limited to, iEos and rEos, and their putative links to tissue homeostasis, host protection, disease activity, and treatment response. Yet fundamental questions remain unresolved and limit translation. Do iEos and rEos represent stable lineages or activation/maturation states within a single lineage? Are they specified in the bone marrow or diversified after tissue entry? Do they function equivalently upon stimulation, and do they share genomic/epigenomic programs? Can they serve as biomarkers to guide the use of monoclonal antibodies in asthma and COPD? Historically, most studies have treated eosinophils as a homogeneous population, which complicates interpretation and cross-study comparisons.

Moving forward, subtype-aware research is essential. We advocate prospective, multi-center studies that (i) predefine iEos and rEos by harmonized cytometric panels (for humans, e.g., Siglec-8, CD62L, CD101, CCR3, CD11b, and CD125) with standardized pre-analytics (anticoagulant, processing time, lysis vs Ficoll), (ii) incorporate single-cell multi-omics (scRNA-seq/CITE-seq, ATAC-seq, proteomics, metabolomics) and spatial profiling in blood and relevant tissues, and (iii) include functional assays (chemotaxis, degranulation, cytokine secretion, phagocytosis) aligned to clinical phenotypes. Longitudinal sampling across stable state, exacerbation, and post-exacerbation is needed to define temporal dynamics and test whether iEos expand during exacerbations in asthma or COPD. In parallel, lineage tracing and bone-marrow progenitor studies should clarify ontogeny and plasticity.

Clinically, the field should evaluate whether iEos frequency (rather than total eosinophil count alone) improves endotyping and treatment selection. Subtype-specific metrics could identify patients with low peripheral eosinophil counts but high iEos proportions who nonetheless display Th2-high biology and may benefit from anti-IL-5/IL-5R strategies. Conversely, patients with airway eosinophilia but low circulating iEos may align better with upstream pathway blockade (e.g., TSLP). Such frameworks require rigorous validation against hard outcomes (exacerbations, lung function, FeNO, symptom scores) and on-treatment trajectories under currently approved biologics.

Finally, we recommend establishing reporting standards for eosinophil-subtype studies (definitions, gating, controls, data sharing) and exploring clinically actionable thresholds for iEos that are reproducible across platforms. Addressing these priorities will refine the biological understanding of eosinophil heterogeneity, enhance patient stratification, and accelerate personalized medicine for eosinophil-associated diseases.

## Data Availability

All data generated or analyzed during this study are included in this published article.
